# Climate Risk Modelling of Balsam Woolly Adelgid Damage Severity in Subalpine Fir Stands of Western North America

**DOI:** 10.1371/journal.pone.0165094

**Published:** 2016-10-25

**Authors:** Kathryn H. Hrinkevich, Robert A. Progar, David C. Shaw

**Affiliations:** 1 Department of Forest Engineering, Resources & Management, Oregon State University, Corvallis, Oregon, United States of America; 2 USDA Forest Service PNW Research Station, Forestry Sciences Lab, La Grande, Oregon, United States of America; Ecole Pratique des Hautes Etudes, FRANCE

## Abstract

The balsam woolly adelgid (*Adelges piceae* (Ratzeburg) (Homoptera: Adelgidae)) (BWA) is a nonnative, invasive insect that threatens *Abies* species throughout North America. It is well established in the Pacific Northwest, but continues to move eastward through Idaho and into Montana and potentially threatens subalpine fir to the south in the central and southern Rocky Mountains. We developed a climatic risk model and map that predicts BWA impacts to subalpine fir using a two-step process. Using 30-year monthly climate normals from sites with quantitatively derived BWA damage severity index values, we built a regression model that significantly explained insect damage. The sites were grouped into two distinct damage categories (high damage and mortality versus little or no mortality and low damage) and the model estimates for each group were used to designate distinct value ranges for four climatic risk categories: minimal, low, moderate, and high. We then calculated model estimates for each cell of a 4-kilometer resolution climate raster and mapped the risk categories over the entire range of subalpine fir in the western United States. The spatial variation of risk classes indicates a gradient of climatic susceptibility generally decreasing from the Olympic Peninsula in Washington and the Cascade Range in Oregon and Washington moving eastward, with the exception of some high risk areas in northern Idaho and western Montana. There is also a pattern of decreasing climatic susceptibility from north to south in the Rocky Mountains. Our study provides an initial step for modeling the relationship between climate and BWA damage severity across the range of subalpine fir. We showed that September minimum temperature and a metric calculated as the maximum May temperature divided by total May precipitation were the best climatic predictors of BWA severity. Although winter cold temperatures and summer heat have been shown to influence BWA impacts in other locations, these variables were not as predictive as spring and fall conditions in the Pacific Northwest.

## Introduction

Invasive species are emerging as one of the most damaging ecosystem disturbances of the twenty-first century [1]. Coupled with the impacts of changing climates, these nonnatives present a substantial threat to biological diversity [2]. The United States spends approximately $120 billion per year managing introduced plants and animals, including the costly impacts associated with impaired ecosystem services [3, 4]. Invasive, nonnative insects pose a particular challenge to management because these insects frequently have no natural enemies and feed on novel hosts that lack defensive abilities. In addition, the life cycles and reproductive success of cold-blooded species are intricately linked to climatic variation [5], and climate suitability largely determines species distributions. Furthermore, the impacts of introduced species can be unpredictable [1], particularly in novel habitats where baseline information is limited. Understanding the relationships between climate and insect-caused disturbances will help to assess infestation risk and to manage impacts, both for current and future climate conditions.

The balsam woolly adelgid, *Adelges piceae* (Ratzeburg) (Homoptera: Adelgidae) (BWA), is a nonnative invasive forest insect introduced to North America around 1900. The insect established and spread in eastern North America, infesting and causing mortality of balsam fir (*Abies balsamea*) in New England and Coastal Canada [6], and Fraser fir (*Abies fraseri* [Purs] Poir.) in the southern Appalachian region of the United States [7, 8, 9]. The adelgid eventually established infestations in all true firs in eastern and western North America, including grand fir (*A*. *grandis*), noble fir (*A*. *procera*), European silver fir (*A*. *alba*), white fir (*A*. *concolor*), Pacific silver fir (*A*. *amabalis*) and subalpine fir (*A*. *lasiocarpa*) in California, Oregon, Washington and British Columbia [10, 11, 12, 13, 14, 15, 16]. Today the insect continues to disperse eastward and northward across Idaho, western Montana and British Columbia [17, 18, 19, 20, 21] where it is causing significant damage to subalpine fir stands. BWA is anticipated to continue to spread throughout the range of subalpine fir and cause significant decline of this important high elevation species.

The life cycle of BWA is comprised of the egg, three nymphal instars and the adult, and reproduction is exclusively parthenogenetic [22, 23]. In the western United States, it typically produces two generations per year [24]: the hemoisistens producing eggs in May following a dormant overwintering period, and the aestivosistens that produce eggs between August and September [6, 25], becoming dormant first instar nymphs (neosistens) in October [26]. Adults oviposit 50 to 200 eggs over a period of approximately 2–10 weeks which can begin emerging after 12 days of incubation, but the timing varies greatly with temperature [6, 27]. Crawlers are the only motile stage, enabling them to find a suitable location to settle either on the original host or through dispersal vectors including birds [28] and wind [20]. Once settled, crawlers insert their stylets into the living host tissue and inject salivary secretions to begin feeding [29]. Within 2–3 days of settling, crawlers become black and secrete a woolly coating of waxy threads that provides protection for all subsequent developmental stages [6].

The timing of BWA development varies directly in response to temperature and survival is linked to the probability of the insects reaching the stage necessary for survival prior to seasonal change [25, 27, 29]. The hemoisistens must enter winter as dormant first instar nymphs (neosistens) in order to survive the cold temperatures [27]. The first instar nymphs of the second generation also undergo a dormant period (summer aestivation) ranging from 2–8 weeks [10], but are less inhibited by cold temperatures and more by summer heat which must remain between a range of approximately 7°C—32°C in order for the nymphs to survive [27]. The fecundity of the spring generation is generally greater than that of the summer generation which must endure the temperature extremes of winter [27]. Reproductive success fluctuates between generations and is mostly influenced by weather, but the condition of the host tree or vigor and fecundity of the insect may also affect population levels [26, 27].

The balsam woolly adelgid can be difficult to evaluate because of its cryptic impacts that vary by geography and host species [23, 25], but there are several common symptoms of an infestation and patterns of damage that indicate severity. Prominent swellings around buds and branch nodes, or gout [6], result from saliva injected into the host tissue while the insect is feeding [23, 29]. Branch gout stunts terminal growth and causes the crown to have a deformed appearance. Severe branch infestations can cause dieback throughout the crown, however, tree decline associated with gout is slow and infestations can persist for many decades before any mortality occurs [30, 31]. A more serious type of attack is a mass stem infestation on the main bole of the tree which can cause severe crown damage, branch dieback and tree mortality in as little as 3 years [13]. Host species vary in their susceptibility to BWA, and environmental conditions such as site quality and water availability that may not directly affect the insect appear to influence the vulnerability of a stand to damage. In the Pacific Northwest, subalpine fir is the most susceptible host species, followed by Pacific silver fir and grand fir, while noble fir and white fir experience only minimal damage [13]. The best sites with the most water availability are often the most severely damaged, presumably because more vigorous trees provide better nutrition for the insect, allowing populations to build rapidly [11, 31]. However, BWA impacts are not consistent, even on a single host within a region, and the factors affecting infestation severity are not well understood [31].

In a previous paper [32], we developed a stand-level damage severity index for BWA based on observed impacts in infested subalpine fir stands. Damage was quantified and described for five discrete severity classes ranging from mildly impacted to severely damaged with high levels of host mortality. In this paper we linked damage severity to climate variability and produced a spatial model of climate-driven BWA risk across the entire range of subalpine fir in the United States. Specifically, our objectives were to 1) identify biologically-relevant monthly and seasonal climate variables that were significantly related to damage severity, 2) build a predictive model to explain variation in observed severity in our study sites, and 3) extrapolate the model across the entire range of subalpine fir to produce a map of climatic susceptibility to BWA for the western United States.

## Materials and Methods

**Fig 1:**
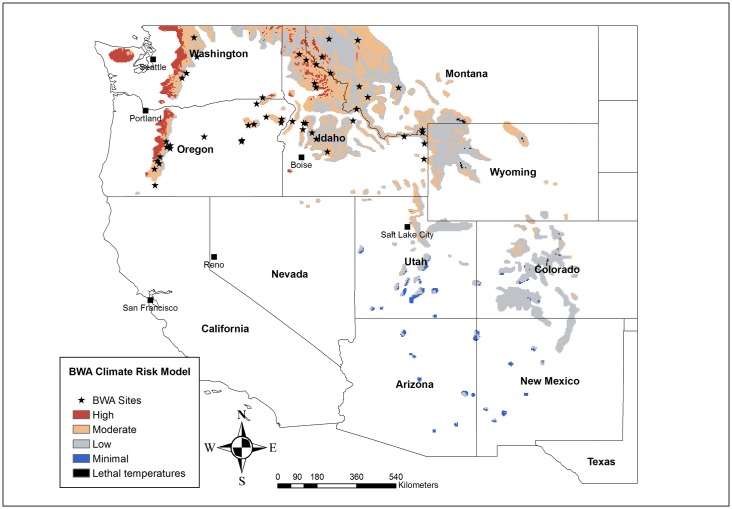
Locations of balsam woolly adelgid (BWA) damage severity sampling sites in California, Oregon, Washington, Idaho, and Montana. Color coding represents model projections of climatic risk for BWA damage for the entire range of subalpine fir in the western United States.

We selected forty-nine subalpine fir stands infested with BWA across the present geographical range of the insect in Oregon, Washington, Idaho and western Montana (Fig 1).

Severity index values were derived using methods designed and tested by the authors for quantifying BWA damage [30]. This index is based on field assessments of the typical symptoms associated with BWA infestations including gout, crown deformity, branch dieback and mortality [6, 13, 30] in the canopy (≥ 12.7cm DBH) and subcanopy (> 10cm height, < 12.7 cm DBH) of affected stands [30]. In this rating system severity is a continuous variable that combines ten standardized metrics into a cumulative score that is positively correlated with stand-level BWA damage. Index values for subalpine fir in the sites sampled were 1.06–5.33, representing stand conditions that ranged from minor branch deformities and little to no mortality to severe crown malformation, branch dieback and subalpine fir mortality up to 75%. Species composition did not directly influence site selection except for a minimum number of subalpine fir (> 10 live trees) required to complete the assessment. Stand types varied from almost pure subalpine fir to mixed conifer containing grand fir, an alternate BWA host, and other non-host species (Table 1). By relative basal area of canopy trees, subalpine fir was generally the dominant species (M = 51.7%, SD = 3.6) comprising between 5.3% and 96.3% of the basal area. Grand fir was only a minor component found in three of the study sites (M = 2.2%, SD = 0.9). Non-host basal areas were dominated by Engelmann spruce, mountain hemlock and lodgepole pine, comprising on average 46.1% (SD = 3.8) of the stands (3.7%–94.7% range). Species composition in the subcanopy was largely dominated by subalpine fir (M = 71.3%, SD = 3.2), with grand fir and non-host species generally comprising a minor proportion of the stems (Table 1).

**Table 1:** Descriptive statistics for stand composition and BWA severity in forty-nine sampling sites. Stand composition is presented in relative values (percentages of total) for basal area in the canopy (≥ 12.7cm DBH) and density in the subcanopy (> 10cm height, < 12.7cm DBH), reported individually for each host species and a combined value for all non-hosts. Severity index values are presented for each host species based on the number of sites in which they occurred.

	N	Mean	Std Dev	Min	Max
Canopy (relative basal area %)					
Subalpine fir	49	51.7	3.6	5.3	96.3
Grand fir	49	2.2	0.9	0	32.5
Non-host	49	46.1	3.8	3.7	94.7
Subcanopy (relative density %)					
Subalpine fir	49	71.3	3.2	23.6	100.0
Grand fir	49	5.5	1.9	0	69.1
Non-host	49	23.3	3.0	0	76.4
BWA severity index values					
Subalpine fir	49/49	2.85	1.00	1.06	5.33
Grand fir	3/49	1.41	0.71	0.59	1.82

We extracted climate records for each site from the PRISM (Parameter–elevation Relationships on Independent Slopes Model) dataset [33], using 30-year normals of average monthly temperatures and precipitation over the most recent three decades (1981–2010). PRISM is a weighted regression technique developed to estimate spatial climate patterns across the counterminous United States, accounting for the physiographic factors that influence climate variation [33,34]. PRISM has been used extensively for climate-related research in mountainous and complex terrain [35]. We downloaded gridded raster datasets (4 km resolution) for monthly temperature (maximum, minimum and mean) and total precipitation from the PRISM website (PRISM Climate Group, Oregon State University, http://prism.oregonstate.edu, created 10 July 2012).

BWA severity index values were regressed on the monthly climate variables using a stepwise procedure to identify significant predictors and build models for comparison. We screened all monthly temperature and precipitation variables individually for significance as well as seasonal and annual averages of each measure. We also derived a new variable calculated as maximum temperature/total precipitation to test the effects of monthly, seasonal and annual hot/dry and cool/wet conditions. This index provides a relative measure of site dryness which has been shown to influence the severity of BWA damage [e.g. 11, 26]. Higher values indicate hot/dry conditions and lower values correspond to cold/wet conditions. Each model was screened for goodness of fit, the portion of variance explained and Akaike Information Criterion (AIC) values [36] to select the strongest model significantly predicting BWA severity.

We calculated regression estimates for each of the 49 sites using the final model equation and used an ANOVA to test whether the model captured significant differences between groups of sites with different severity levels (defined below). Welch’s testing for significance accounted for unequal variances between sample groups. We modified the original five-class damage severity rating system [32] into two groups to emphasize the differences between severely damaged stands (group 2) and those with fewer impacts (group 1). Group one included sites with index values below 3.00, representing stands with little to no host mortality [32]. Group two included index values of 3.00 and higher, representing stands that incurred moderate to high levels of BWA-caused damage mortality [32]. Dieback, gout and crown malformation were noticeably more severe in group two, with stands averaging greater than 50% branch dieback, moderate to severe branch deformity and multiple crown symptoms including drooping leaders and stunted terminal or lateral branches. We generated summary statistics of the regression estimates for the two groups to define risk categories for mapping. The categories included minimal, low, moderate and high risk groups, which represented the non-overlapping ranges between minimum and maximum values for groups one and two.

To produce the final risk map that extrapolates the data beyond the measured locations we clipped the climate raster layers for each variable included in the regression model to the spatial extent of subalpine fir distribution in the United States [37]. We calculated regression estimates for each raster cell and assigned each cell to one of the four BWA risk categories described above. All cells with January mean temperatures < 11°C and summer (June, July, August) average monthly maximum temperatures > 32°C were reclassified as lethal temperature zones for infestation development based on temperature-mortality relationships established in previous studies [27, 38]. Although areas prone to experiencing lethal temperatures may maintain low populations levels of surviving insects, 30-year climate trends exceeding these temperature thresholds in monthly averages suggest that an area experiences consistent climatic suppression and does not likely fit our severity estimation model generated from sites that were not exposed to these temperature extremes.

## Results

The significant individual predictors from the stepwise regression showed seasonal patterns in their relationship with damage severity (Table 2). Late summer and early fall (August through October) explained the most variance and September minimum temperature was the best predictor overall. The August relationship was nearly identical to that of September. All fall and late summer relationships with minimum temperature were positive. Monthly minimum temperatures were also explanatory for winter (December through February), but the relationships were not as strong as those in the late summer and fall. Maximum temperatures significantly predicted damage severity in early summer (June and July) and the relationship was negative. Our dryness index (maximum temperature/total precipitation) was only significant for May and negatively related to the damage index. There were no significant relationships with precipitation or with annual climate averages.

**Table 2:** Summary of significant climate models tested and the corresponding statistics for each. Tmin, Tmax and Ppt represent average minimum temperature, maximum temperature and total precipitation for 30-year climate normals (1981–2010). All significant results for individual months and seasonal averages are shown. B1 and B2 show regression coefficients for the first and second, if present, independent variable. The final model is shown in bold.

Model	B1	B2	p-value	R^2^	AIC
*Winter (Dec-Feb)*					
Dec T_min_	0.13	.	0.0313	0.09	-1.77
Jan T_min_	0.12	.	0.0275	0.10	-2.00
Feb T_min_	0.13	.	0.0291	0.10	-1.90
Winter T_min_	0.13	.	0.0286	0.10	-1.93
*Spring (March-May)*					
May T_max_ / May P_tot_	-0.20	.	0.0465	0.08	-1.33
*Summer (June-Aug)*					
June T_max_	-0.02	.	0.0348	0.09	-1.58
July T_max_	-0.18	.	0.0418	0.09	-1.25
Summer T_max_	-0.19	.	0.0455	0.08	-1.10
July T_min_	0.20		0.0421	0.09	-1.23
Aug T_min_	0.23		0.0117	0.13	-3.57
Summer T_min_	0.20		0.0499	0.08	-0.93
*Fall (Sept-Nov)*					
Sept T_min_	0.22	.	0.0106	0.13	-3.76
Oct T_min_	0.23	.	0.0143	0.12	-3.21
Nov T_min_	0.16	.	0.0362	0.09	-1.50
Fall T_min_	0.22	.	0.0137	0.12	-3.28
*Annual*	.	.	.	.	
*Model comparisons*					
July T_min_ + (May T_max_ / May P_tot_)	0.25	-7.94	0.0039	0.21	-6.68
Aug T_min_ + (May T_max_ / May P_tot_)	0.24	-7.14	0.0021	0.24	-8.03
**Sept T**_**min**_ **+ (May T**_**max**_ **/ May P**_**tot**_**)**	**0.24**	**-7.26**	**0.0017**	**0.24**	**-8.46**
Oct T_min_ + (May T_max_ / May P_tot_)	0.22	-5.91	0.0068	0.20	-5.51

We tested various combinations of minimum temperature and the May dryness variable in our multiple regressions, focusing on the period between late summer and early fall. The final model we selected was 3.134 + 0.236 * September minimum temperature– 7.264 * (May maximum temperature/May total precipitation). The model significantly predicted severity index values (R^2^ = 0.24, F (2,46) = 7.34, p = 0.0017, and both September minimum temperature (t (48) = 3.07, p = 0.0106) and the May dryness variable (t (48) = -2.60, p = 0.0126) were significant. This model explained the most variance and had the largest AIC value, however, the August model was nearly identical. Multicollinearity between independent variables prevented us from choosing more than one monthly temperature predictor, of which September was the best predictor. Seasonal averages also explained less variance in damage severity than the single month September predictor, especially when paired with the May dryness variable.

Regression estimates were significantly different for the two damage severity groups (F(1,44) = 12.975, p = 0.0008. Group one (n = 27, M = 2.7, SD = 0.5) had significantly lower regression estimates than group two (n = 22, M = 3.1, SD = 0.3). Group one model estimates ranged from 1.6–3.5 and group two ranged from 2.6–3.8, defining the boundaries of the four risk categories, minimal, low, moderate and high (Table 3).

**Table 3:** Predictor variable and regression estimate ranges for the BWA damage risk categories identified in the ANOVA analysis. Tmin, Tmax and Ppt represent average minimum temperature, maximum temperature and total precipitation for 30-year climate normals (1981–2010). Risk category values are defined by the non-overlapping ranges of regression estimates for the low damage (1.6–3.5) and severe damage (2.6–3.8) groups.

Risk category	September Tmin (°C)	May Tmax (°C)	May Ppt (mm)	May Tmax (°C) / May Ppt (mm)	Regression estimate
Minimal	< -0.1	> 15.8	< 43.4	> 0.3	< 1.6
Low	-0.1–1.8	15.5–15.8	43.4–53.7	0.2–0.3	1.6–2.6
Moderate	> 1.8–6.4	11.4–< 15.5	> 53.7–118.2	0.1–< 0.2	> 2.6–3.5
High	> 6.4	< 11.4	> 118.2	< 0.1	> 3.5

The mapped spatial variation of risk classes for subalpine fir (Fig 1) indicates a gradient of climatic susceptibility generally decreasing from the Olympic Peninsula in Washington and the Cascade Range in Oregon and Washington eastward, with the exception of some high risk areas in northern Idaho and western Montana. There is also a pattern of decreasing susceptibility from north to south. The east slope of the Cascade Range, the Blue Mountains in Oregon, and the Rocky Mountains from Wyoming through Idaho, Montana and Washington all have regions of moderate susceptibility. Subalpine fir stands to the south of Wyoming in Utah, Colorado, Arizona and New Mexico are at minimal to low climatic risk for BWA damage. Regions experiencing lethal temperatures were restricted to the eastern edge of the subalpine fir distribution in Colorado, Wyoming, and south-central Montana.

## Discussion

Balsam woolly adelgid damage severity is significantly influenced by spring and fall climatic conditions in the Pacific Northwest. Specifically, warmer September minimum temperatures combined with cool and wet May conditions were associated with higher severity BWA impacts. An almost equal alternate model for August and May dryness suggests that the importance of fall minimum temperature likely begins in August. Both of these relationships are consistent with the biology and life cycle of the insect. These are crucial time periods in the timing of host selection and oviposition for the aestivosistens (summer generation) which occurs in August through late fall, and in the development of the hiemosistens (overwintering generation) which begins in August/late fall and ends with oviposition the following May [6, 29].

These finding differ from previous research which has emphasized the importance of summer and winter temperature extremes as drivers of BWA damage severity [27, 38]. It is not surprising, however, given the milder climate of the mountainous west in comparison to southeastern Canada and the northeast United States where most of the existing climatic relationships with extreme temperatures were established. Western subalpine fir environments rarely experience the temperature extremes of eastern North America which effectively restrict the dispersion and survival of BWA. In the absence of these strict climatic controls, BWA infestation severity would reasonably be influenced more by subtle climate variations that are not lethal.

The importance of fall temperatures has been indirectly established in the context of overwinter mortality. Adelgids must enter winter as first instar nymphs that have reached dormancy with their stylets inserted into the tree to be successful [6]. Overwinter survival in New Brunswick has been shown to be inversely related to the coldest temperatures, however, hiemosisten mortality depended on both the duration of exposure to low temperatures and the date of exposure, not a fixed temperature [27]. That link between unseasonal low temperatures occurring before the insect has cold-hardened for the winter and high mortality directly supports our results for September minimum temperatures, even in the absence of lethal cold events during dormancy. Warmer Septembers may increase survival rates by ensuring timely development before the onset of winter, with greater reproductive success promoting more population growth and more severe damage to the stand.

In our model, spring temperature and precipitation better predicted BWA severity than summer heat, presumably because these mountainous sites do not experience summer extremes capable of causing population decline. That our highest severity occurred with cool, wet May conditions may be attributed to the influence of climate on host suitability. Subalpine fir growth rates have been found to be the highest in cool, wet May/June conditions [39], and vigorous tree growth has been associated with higher quality hosts capable of supporting larger BWA populations [11, 23]. This hypothesis is supported by previous observations in Oregon and Washington demonstrating that most BWA damage to subalpine fir occurred in mesic environments that encouraged the best tree growth [40]. Similar observations were made for Pacific silver fir in Washington [11]. Direct effects of spring climate on the summer generation are likely less important than indirect effects on host suitability or preference, which explains the higher fecundity of the spring generation compared to the summer generation that must endure overwintering temperatures [27]. Although temperature has been found to influence developmental duration of aestivosistens in the laboratory, field development varies between 4–7 weeks, suggesting that the physiological condition of the tree has a pronounced effect on fecundity, confounding the effects of temperature [27].

Our predictive map indicates that subalpine fir on the Olympic Peninsula and in the western Cascade Mountains in Oregon and Washington had the most extensive high risk areas, with northern Idaho and Montana having many mountain ranges with potential for high severity. The central and southern Rocky Mountain habitat of subalpine fir does not appear to be particularly vulnerable, particularly the regions to the east and south of Idaho. This could be explained by very droughty conditions typical of the spring and summer months in these regions, which, when combined with the characteristic low fall and winter temperatures, minimizes climatic susceptibility according to our model. The location of lethal temperature zones exclusively at the eastern edge of the subalpine fir range is consistent with the west to east trend of decreasing risk. That no lethal temperature zones occurred west of the Idaho/Wyoming border supports our interpretation of subtle spring and fall conditions as the dominant climatic controls on BWA damage severity in the western United States.

A note of caution needs to be expressed recognizing the potential bias resulting from BWA impacts that may not have been fully realized in our study sites, or from unexpected consequences in naive ecosystems yet to be exposed, particularly outside of the Pacific Northwest where the model results were extrapolated. This will likely always be the case with introduced species and range expansion scenarios into novel habitat, and is therefore a factor we attempted to control for with sampling design, but did not entirely eliminate. We focused on a single host species, subalpine fir, at high elevations (961–2288 meters) exclusively in the Pacific Northwest where the insect is currently active. Slight differences in community composition may influence our extrapolation beyond this region, however, the habitat preferences of subalpine fir should provide enough consistency to model climatic relationships for this species across the mountainous west. Regarding the presence of grand fir, an alternate host for BWA in the Pacific Northwest, as a potential confounding factor in the model, differences in host susceptibility between the two species should minimize any influence this may have had on the extrapolation. Grand fir is one of the most resistant hosts to the adelgid [13], particularly at elevations above 1000 meters where little damage has been observed even when found adjacent to heavily infested subalpine fir [32, 40]. Although grand fir did co-occur with subalpine fir in three of our sites, the targeted sampling in high elevation communities (M = 1703 meters, SD = 290) and low number of instances (3 sites) should have limited any substantial influence on the model predictions. Despite these limitations, modeling climatic susceptibility to BWA using quantitative damage severity data shows promise as a method for improving our understanding of the patterns of infestation severity and potential risks associated with range expansion. The model can be refined with ongoing efforts to expand the network of study sites using the same damage severity index used here in order to maintain consistency in the stand assessments. The approach could also be extended to include other *Abies* species which will provide much needed information about the varied impacts on each host. Model strength would also be improved by incorporating environmental variables such as slope, aspect, elevation and forest stand data such as host density, age, size and overall species composition into the predictions. Spatial risk modelling such as this will provide a powerful tool for decoupling the complex interactions that influence BWA damage severity and aid land managers in their efforts to protect valuable ecosystems.

This study provides an initial step in modeling the climatic susceptibility of BWA damage across the range of subalpine fir in the western United States. Our findings integrate a damage severity assessment protocol [32] with measurable climate variables to quantitatively assess the relationship between BWA impacts and climatic variation. This is the first model to use a quantitative damage metric other than mortality or presence/absence data, and is an important step for extending monitoring efforts into a modelling framework for the western United States.

## Supporting Information

S1 File Supplemental data file. (CSV)
